# Is There a Strength Deficit of the Quadriceps Femoris Muscle in Patients Treated Conservatively or Surgically after Primary or Recurrent Patellar Dislocations? A Systematic Review and Meta-Analysis

**DOI:** 10.3390/jcm13175288

**Published:** 2024-09-06

**Authors:** Carlo Biz, Pietro Nicoletti, Mattia Agnoletto, Nicola Luigi Bragazzi, Mariachiara Cerchiaro, Elisa Belluzzi, Pietro Ruggieri

**Affiliations:** 1 Orthopaedics and Orthopaedic Oncology, Department of Surgery, Oncology and Gastroenterology DiSCOG, University of Padova, Via Giustiniani 3, 35128 Padova, Italy; carlo.biz@unipd.it (C.B.); mariachiara.cerchiaro@unipd.it (M.C.); pietro.ruggieri@unipd.it (P.R.); 2 Centre for Mechanics of Biological Materials, University of Padova, 35131 Padova, Italy; 3 Department of Neurosciences, University of Padova, 35128 Padova, Italy; pietronicoletti.ft@gmail.com (P.N.); mattia.agnoletto.2@studenti.unipd.it (M.A.); 4 Human Nutrition Unit (HNU), Department of Food and Drugs, University of Parma, 43125 Parma, Italy; robertobragazzi@gmail.com; 5 Musculoskeletal Pathology and Oncology Laboratory, Department of Surgery, Oncology and Gastroenterology DiSCOG, University of Padova, Via Giustiniani 3, 35128 Padova, Italy

**Keywords:** dynamometer, isokinetic, muscle strength, patellar dislocation, quadriceps femoris

## Abstract

**Background:** Patellar dislocation is a knee injury affecting generally young, active individuals, damaging joint ligaments and structures, and impacting sports activity and quality of life. **Objective:** This review aimed to evaluate the role of the quadriceps femoris muscle in knee extension and to consider whether extensor strength deficits are present in patients who have suffered from a primary or recurrent patellar dislocation and have been treated surgically or conservatively. **Methods:** This systematic literature review with meta-analysis was performed following the PRISMA Statement criteria. The search engines consulted to select studies were MEDLINE/PubMed, Scopus, and Web of Science/ISI. The JBI Critical Appraisal Checklist tools were applied for the quality assessment based on the specific study design. The outcomes were measurements of the knee extension force of the quadriceps femoris muscle, which were objectively quantifiable with an isokinetic or mobile dynamometer. **Results:** Of the 891 articles initially identified through the databases, 10 studies with a total of 370 patients were included in the analysis. The results indicated a strength deficit of the quadriceps in patients who had undergone a patellar dislocation, in comparison with the control group, when examining the uninvolved limb or in comparison with the pre-operative values. The overall effect size was large, with a value of −0.99. **Conclusions:** Our review concluded that after a primary or recurrent patellar dislocation, strength deficits of the quadriceps femoris muscle in the knee extension of the affected limb are frequently observed in surgically or conservatively treated patients. This deficit may persist even after a protracted follow-up of up to three years after injury.

## 1. Introduction

Patellar dislocation, which accounts for approximately 3% of all knee injuries, is characterized by the loss of contact between the articular surfaces of the patella and the femoral trochlea, with the dislocation being predominantly lateral in nearly all cases [1,2]. This injury is often associated with osteochondral and chondral fractures of the patellar and femoral condyle facets, as well as damage to the ligaments of the medial compartment, particularly the medial patellofemoral ligament (MPFL) and the medial retinaculum [1,3,4,5,6,7,8]. Over time, acute patellar dislocations can lead to patellar instability, recurrent dislocations, pain, diminished knee function, increased sports-related disability, patellofemoral osteoarthritis, and reduced quality of life [9,10,11].

The incidence in the general population is 5.8 per 100,000 individuals, with a higher rate of 29 per 100,000 in the 10- to 17-year age group, particularly among young and active individuals due to trauma during sports activities. The incidence is comparable between genders [3,12,13]. At the time of injury, the foot is planted on the ground while the individual executes a shearing motion in the opposite direction. This movement causes internal rotation of the femur, external rotation of the tibia, and a valgus knee position [14]. The quadriceps muscle shortens, exerting a lateral force on the patella, typically during the initial phase of knee flexion [15,16].

A partial or complete rupture of the MPFL, which serves as the primary static restraint against lateral patellar instability, is detectable in 97% of lateral patellar dislocation cases [4,17]. Other structures, such as the superficial medial collateral ligament (sMCL) and the medial meniscal ligament (mML), may also be injured [18]. Additionally, bone edema or osteochondral lesions on the patellar articular surface can lead to intra-articular fragments [5]. Anatomical abnormalities in the patellofemoral joint position increase the risk of patellar instability and dislocation [17,18,19,20,21,22,23].

There is no consensus on the treatment of primary patellar dislocation, with conservative management typically recommended as the initial approach unless there are bone fragments, severe intra-articular damage, or significant injury to the patellar ligament complex [4,11,23,24,25,26]. Various surgical techniques are available, with repositioning of the medial ligamentous compartment being the most commonly performed procedure [16,18,20,27,28,29,30]. Physiotherapy plays a crucial role in restoring the full range of motion (ROM) and strengthening the quadriceps femoris muscle to re-establish the dynamic stability of the patellar soft tissue [11,31]. Therapeutic exercise is considered essential at every stage of recovery; however, there is a lack of specific guidelines regarding the exercises or parameters to be used in conservative treatment [30,31,32].

This review aimed to investigate the quadriceps strength deficit during knee extension in individuals who have experienced primary or recurrent patellar dislocation, whether treated conservatively or surgically, through a systematic review of the literature and meta-analysis. The outcomes assessed included knee extension force values, objectively measured by a mobile or isokinetic dynamometer and compared to healthy knees to determine the presence of extensor strength deficits in the quadriceps.

## 2. Materials and Methods

### 2.1. Search Strategy

This systematic review and meta-analysis were conducted in accordance with the guidelines outlined in the “Preferred Reporting Items for Systematic Reviews and Meta-Analyses” (PRISMA-2020) framework [33], which was used to guide and monitor the research process. The study protocol has been registered within the Open Science Framework repository (Identifier code: DOI: 10.17605/OSF.IO/N8VPJ).

A comprehensive literature search was conducted across three major databases: Scopus [34], Web of Science (WoS)/ISI [35], and MEDLINE/PubMed [36], with searches performed between May and June 2023. Keywords were carefully selected and combined using Boolean operators to focus on topics related to patellar dislocation and lower limb strength. The search string utilized was: “patellar dislocation” AND (“muscle strength” OR “muscle weakness” OR dynamometer OR isokinetic OR isometric OR isotonic OR eccentric OR concentric OR strength OR torque OR power OR force). Filters were applied to include studies published between 1 January 2003 and 30 September 2023, ensuring the inclusion of the most recent research on both conservative and surgical treatments.

### 2.2. Inclusion Criteria

The search was limited to articles written in English and classified as original articles, covering a span of 20 years. The inclusion criteria focused on randomized clinical trials (RCTs), cohort studies, case-control studies, and case series that met the following PICOS criteria:

- Population: Patients with primary or recurrent patellar dislocation, whether traumatic or atraumatic, male or female, aged 14 years or older.
- Intervention: The study included conservative interventions involving muscle strengthening or surgical treatments aimed at patella stabilization.
- Controls: Evaluations were conducted before and after the intervention, utilizing a homogeneous control group. This control group could consist of healthy subjects, a comparison with the contralateral limb of the same individual, or a comparison between two different types of interventions.
- Outcome: The primary outcome was the measurement of the knee extension force of the quadriceps, which was objectively quantified using an isokinetic or mobile dynamometer.

### 2.3. Exclusion Criteria

Articles that included participants with anterior knee pain, patellofemoral pain, or other knee disorders—whether isolated or in combination with patellar dislocation—were excluded from consideration. Additionally, studies involving individuals with a history of prior patellar injuries were also not considered. Furthermore, all pediatric-age dislocations were excluded due to significant anatomical and physiological differences in this age group. Children are still undergoing musculoskeletal development, with active growth plates and changes in bone and ligament structures, leading to different injury mechanisms and treatment responses compared to older adolescents and adults. Additionally, the causes and treatment protocols for patellar dislocations in children often differ, necessitating a separate analysis to ensure accurate conclusions. By focusing on individuals aged 14 and older, the present study targets a more homogeneous population with similar anatomical and functional characteristics, thereby enhancing the relevance and reliability of the findings. 

Studies were excluded if they did not employ objective, instrument-based force measurements or if the data were not quantitative or unavailable. Additionally, articles published before 1 January 2003, those not written in English, as well as clinical cases, case reports, interviews, book chapters, opinion pieces, commentaries, narrative reviews, systematic reviews, meta-analyses, and articles without accessible full texts, were excluded from this review.

### 2.4. Study Selection

Following the PRISMA-2020 guidelines, the primary objective of this procedure was to identify and evaluate studies suitable for inclusion in the review. After removing duplicates from the initial database search, two independent reviewers (MA and PN) screened the titles and abstracts of all retrieved publications and conducted a full-text assessment for potential inclusion. Any disagreements between the reviewers were resolved through discussion, with consultation from a third reviewer (CB) if necessary. The inter-rater agreement was high, with kappa statistics of ≥0.83, indicating strong concordance between the reviewers.

### 2.5. Outcome Measures

To be included in this review, studies were required to provide objective measurements of quadriceps muscle strength during knee extension. These measurements could be obtained using either an isokinetic or a handheld/mobile dynamometer. The studies could vary in their approach, utilizing different knee flexion angles when using handheld dynamometers and employing various angular velocities with isokinetic dynamometers to quantify the knee extension force.

### 2.6. Data Extraction

All relevant data from the included studies were systematically extracted and recorded in an Excel file. The extracted information included the author, publication date, study design, participant groups, number of patients, sex/gender, age, number of patients lost to the follow-up, duration of follow-up, type of dynamometer used, and various outcome measures related to quadriceps muscle strength in knee extension. Two reviewers (MA and PN) independently performed the data extraction. Any discrepancies between the reviewers were resolved through discussion or, if necessary, with the assistance of a third reviewer (CB).

### 2.7. Quality Assessment 

To account for the heterogeneity in study design and methodology among the selected studies, the Joanna Briggs Institute (JBI) Critical Appraisal tools were employed to critically assess their quality for inclusion in this systematic review. The JBI tools offer tailored checklists based on the specific study designs, evaluating various quality criteria. Each item on the checklist is rated with one of four possible responses: “yes”, “no”, “unclear”, or “not applicable”.

### 2.8. Statistical Analysis and Meta-Analysis

A meta-analysis was conducted using Prometa3 software (Internovi, Cesena, Italy, 2024). Effect sizes (ESs) were calculated as standardized mean differences between the control and intervention groups, utilizing average values (means) and standard deviations (SDs) unless only medians with interquartile ranges (IQRs) were provided. In such cases, the median and IQR were converted to mean and SD using the transformation method proposed by Wan et al. [37]. When the 95% confidence intervals (CIs) were provided instead of SDs, these were estimated using the following formula:

$$SD=\frac{\sqrt{N}\;\cdot \;\left(upper\;limit-lower\;limit\right)}{3.92}$$

If the data were from the contralateral limb of the same individual, a correlation coefficient of 0.70 was imputed.

The 95% CIs for each ES were calculated following the approach of Hedges and Olkin [38]. According to Cohen’s guidelines, the magnitude of the computed ES was interpreted as follows: small = 0.20, moderate = 0.50, and large ≥ 0.80.

A fixed-effect model was applied when the heterogeneity among studies was nonsignificant based on the I^2^ statistic; otherwise, a mixed-effect model was used. ESs were calculated for each study included in the meta-analysis, and an overall ES was determined, incorporating the weight of each study. If a study reported different but comparable outcome measures, these were combined using a correlation coefficient of 0.70 to account for their relationship. Finally, forest plots were employed to visualize the computed ESs.

Publication bias was evaluated through a visual inspection of the funnel plot.

## 3. Results

### 3.1. Selection of Studies

**Figure 1:**
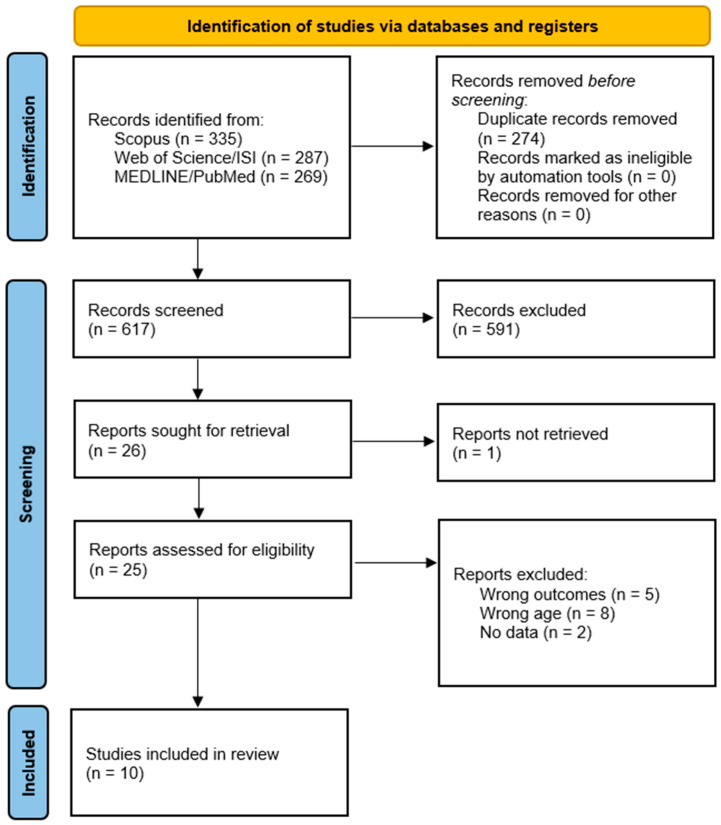
Flowchart of the search strategy conducted in compliance with the criteria outlined in the “Preferred Reporting Item for Systematic Reviews and Meta-Analyses” (PRISMA) guidelines [33].

The initial search yielded 891 studies, with 335 identified from Scopus [34], 287 from Web of Science/ISI [35], and 269 from MEDLINE/PubMed [36]. After removing 274 duplicate articles, 617 unique studies remained. Of these, 458 were excluded for being off-topic, and 115 were excluded because they were systematic reviews, narrative reviews, or case reports. Additionally, 18 articles were excluded for not being written in English. Following this initial screening, 26 eligible articles remained for further analysis. Upon closer examination of these 26 articles, 5 were excluded for analyzing outcome measures other than quadriceps strength, 8 were excluded for including subjects under 14 years of age, and 2 were excluded for not presenting quantitative data. One report was excluded as it was not retrieved. Ultimately, 10 studies met the inclusion criteria and were included in this review [39,40,41,42,43,44,45,46,47,48]. The screening process is detailed in Figure 1.

### 3.2. Population Characteristics 

Among the ten studies [39,40,41,42,43,44,45,46,47,48] reviewed, 370 participants were reported (40.9% male, 59.1% female). The mean age of the patients was 23.4 years. Other general characteristics are represented in Table 1.

### 3.3. Quality Assessment

The quality analysis results were as follows: in the Case Series Tool, Woods et al. [48] reached 9/10, and Ronga et al. [44] 8/10; in the Case Control Tool, Mikashima et al. [43] reached 6/10, Smith et al. [45] 4/10, Tompkins et al. [47] 8/10, Asaeda et al. [40] 8/10, Keilani et al. [41] 7/10; in the RCT Tool, Smith et al. [46] scored 13/13, the highest score reached in this review; finally, in the Cross-Sectional Tool, Arrebola et al. [39] reached 7/8 and Lucas et al. [42] 5/8. All items are shown in Table 2.

### 3.4. Type of Intervention and Follow-Up

The groups and the different interventions performed in the included studies are reported in Table 1. Surgical [40,41,43,44,45,47,48] or conservative [46] interventions were performed. A few studies [39,42] did not report any intervention. In some studies, a comparison was carried out with the contralateral limb of the same subject [44,47,48], while in the remaining studies, a control group was set considering an uninjured population [39,42,46] or two different types of intervention were compared [41,45], or post-surgical values were compared to pre-operative ones [40,45]. In addition, the number of patients who completed a follow-up period expressed in months is reported in Table 1.

### 3.5. Outcome Measures

The studies reviewed in the present systematic review focused on evaluating the outcome measures related to knee extension force, specifically targeting the quadriceps muscle. Two types of dynamometers were employed across the studies: isokinetic in three studies [41,44,48] and mobile in seven studies [39,40,42,43,45,46,47]. The assessments involving the mobile dynamometer were conducted at varying degrees of knee flexion, while the isokinetic dynamometer measurements were taken at different angular velocities. As a result, the units of measurement varied across the studies. Detailed information on the methodology and measurement parameters is provided in Table 3. 

### 3.6. Statistical Analysis and Meta-Analysis

In this systematic review, a meta-analysis of the data could be performed for eight out of the ten studies retrieved. The studies by Mikashima et al. [43] and Keilani et al. [41] were not included in the meta-analysis because the data provided were incomplete or insufficient for meta-analytical computations. Twenty-two ESs could be computed, as shown in Table 4.

**Figure 2:**
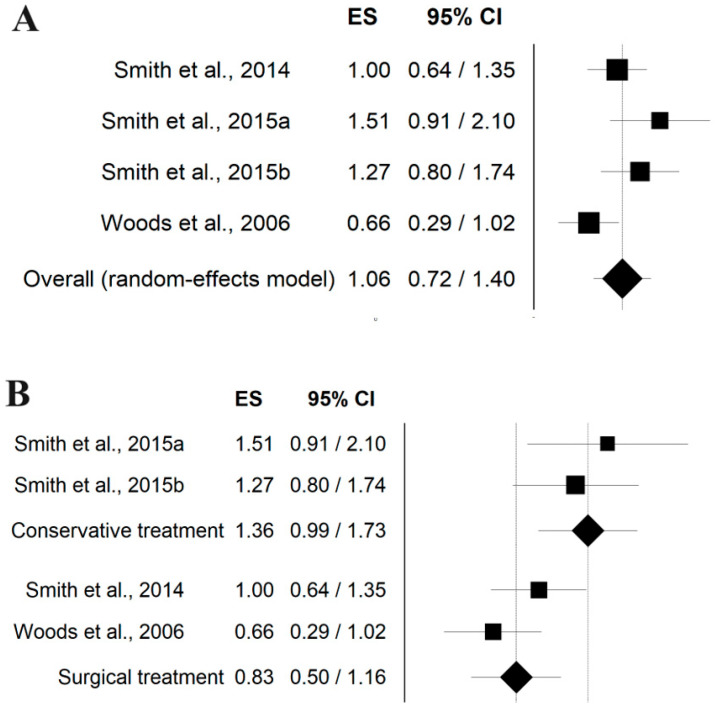
Forest plot of the studies assessing the recovery in quadriceps strength post intervention overall (**A**) and stratified based on the type of intervention—conservative or surgical (**B**) [45,46,48].

Three studies [45,46,48] assessed the recovery in quadriceps strength post-intervention, either conservative [46] or surgical [45,48], finding a large effect. The overall ES was computed at 1.06 [95% CI 0.72–1.40] (Figure 2A). Stratifying based on the type of intervention, the ES for conservative treatment was 1.36 [95% CI 0.99–1.73], suggesting a positive effect on quadriceps strength. Surgical treatment had a lower ES of 0.83 [95% CI 0.50–1.16] (Figure 2B).

**Figure 3:**
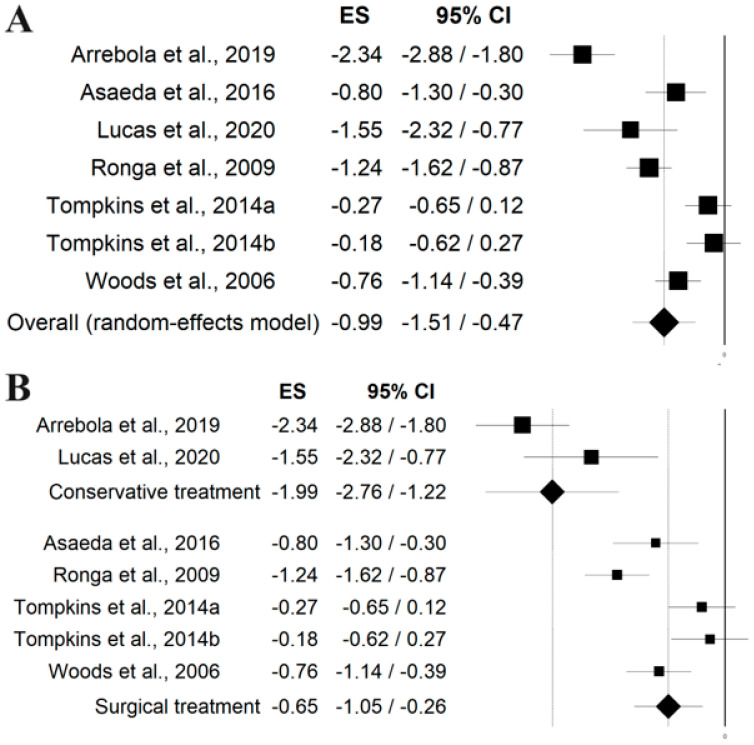
Forest plot of the studies assessing the residual deficit in quadriceps strength post-intervention compared to a control sample or to an unaffected contralateral limb, overall (**A**) and stratified based on the type of intervention—conservative or surgical (**B**) [39,40,42,44,47,48].

Six studies [39,40,42,44,47,48] assessed outcome measures concerned the strength of the quadriceps objectively quantified with an isokinetic or mobile dynamometer to test the effect of an intervention and the recovery of strength in patients compared to a control sample or to an unaffected contralateral limb. The overall ES was −0.99 [95% CI −1.51 to −0.47], indicating a residual deficit in quadriceps strength post-intervention (Figure 3A). Stratifying based on the type of intervention, the deficit was larger, with conservative treatment having an ES of −1.99 [95% CI −2.76 to −1.22]. Conversely, surgical treatment showed a smaller residual deficit, with an ES of −0.65 [95% CI −1.05 to −0.26] (Figure 3B). 

**Figure 4:**
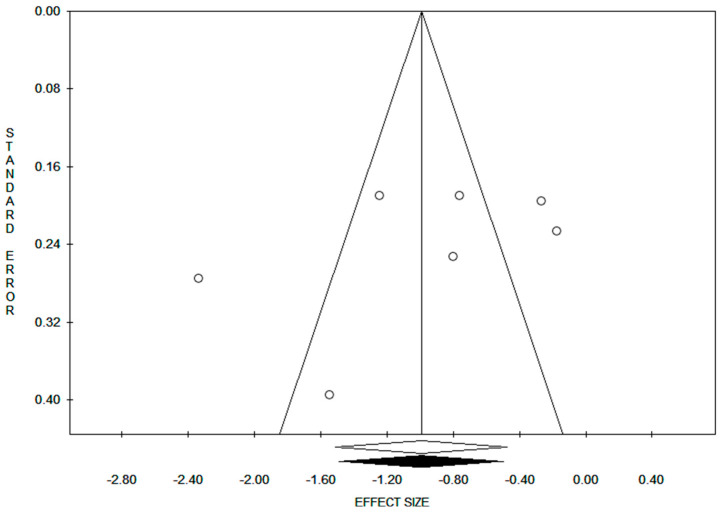
Funnel plot showing no evidence of publication bias.

Visual inspection of the funnel plot (Figure 4) did not detect publication bias.

## 4. Discussion

This systematic review with meta-analysis aimed to evaluate quadriceps muscle strength in knee extension, objectively quantified using a mobile or isokinetic dynamometer in patients who have experienced a primary or recurrent patellar dislocation, treated either surgically or conservatively. The mean age of participants was 23.4 years, characterizing a young population as supported by epidemiological data [2,3,7,9,12]. Follow-up periods varied significantly across the studies, ranging from a few months post intervention to up to three years. Effective treatment for primary patellar dislocation requires investigations that extend beyond the mere assessment of recurrent injury incidence, as patients frequently experience residual symptoms of instability or pain that significantly impair their quality of life and ability to return to sports, even in the absence of further dislocations [4,9,49,50].

Patellar dislocations can be managed through either conservative or surgical approaches; the studies included in the present review showed that both conservative and surgical interventions had a positive effect on quadriceps strength recovery, with an overall ES of 1.06 indicating a substantial improvement in strength post-intervention, which is a promising finding for patients with patellar instability undergoing these treatments. Conservative treatments, such as physical therapy, rehabilitation exercises, or other non-invasive methods, may result in a more pronounced initial improvement in quadriceps strength, with an ES of 1.36. This large effect could be due to the focused, intensive nature of rehabilitation programs that emphasize muscle strengthening. The lower ES of 0.83 for surgical interventions might indicate that while surgery can restore function, the recovery of muscle strength might be less pronounced compared to conservative methods. This could be due to the fact that surgery often involves trauma to the tissue, leading to a longer recovery period before strength gains are fully realized.

Quadriceps strengthening is important to achieve and maintain proper stabilization of the knee joint [51,52]. Some authors have investigated whether a strengthening program specific to the Vastus Medialis Oblique (VMO), a crucial medial stabilizer of the patellofemoral joint [10], could reduce quadriceps deficits. Results from Smith et al. [46] indicated promising findings of interventions targeting VMO, with ESs in the range of 1.27–1.51. However, other studies have shown that selectively recruiting quadriceps fibers over others is not supported by current evidence [53,54,55,56]. Consequently, it has been proposed in some studies that quadriceps strengthening should be accompanied by reinforcing the core and hip muscle complex, and improving neuromotor control [11,31]. Strengthening these muscles is considered significant for a safe return to sports because, during dynamic activities, they absorb external moments at the hip and knee by contracting eccentrically [57,58,59]. For instance, in monopodal landing, reduced hip muscle complex strength has been associated with increased knee valgus in healthy women [60], whereas reduced quadriceps strength has been linked to decreased knee flexion in patients undergoing anterior cruciate ligament reconstruction [61]. Since these movement patterns, particularly knee valgus and internal hip rotation, are implicated in the injury mechanism of patellar dislocations [14,15], some studies [57,58,59,60,61] have suggested that restoring lower extremity strength may improve knee joint stability and reduce the risk of re-injury.

Additionally, increased quadriceps strength may protect the patellofemoral joint from cartilage deterioration [62], which is particularly important as patients who suffer from patellar dislocations have an increased risk of developing patellofemoral osteoarthritis [63]. Only a few systematic reviews in the literature [64,65] have investigated lower limb strength recovery in patients who have suffered at least one patellar dislocation. These reviews confirm the findings of our review, highlighting the frequent presence of incomplete recovery and persistent deficits in knee extension strength. Smith et al. [65] focused on clinical outcomes post-rehabilitation, excluding surgically treated patients, and found that the Muscle Power Scale (MRC) scoring used to test patient strength, ranging from 0 to 5, was inadequate for detecting strength deficits due to its lack of accuracy [66].

On the other hand, recovery after conservative methods is often incomplete, potentially affecting sports participation and overall quality of life [9,67]. Our systematic review revealed a large residual deficit in quadriceps strength after conservative treatment, with an ES of −1.99. In the studies by Arrebola et al. [39] and Lucas et al. [42], patients who did not undergo surgical interventions exhibited significant quadriceps strength deficits of 43.7% and 39.1%, respectively, when compared to uninjured controls, with statistically significant ESs of −2.34 and −1.55. This could imply that while conservative treatments may offer greater immediate gains, they might not sustain those gains over time, or they may not address underlying structural issues that could impede full recovery. 

Conversely, the smaller residual deficit (ES of −0.65) following surgical treatment seems to suggest that surgery, while perhaps leading to smaller initial gains, may provide a more stable or sustained recovery in the long term. The surgical repair of damaged structures might offer a better foundation for regaining strength, even if the initial recovery process is slower. In studies where MPFL repair was performed, such as in Ronga et al. [44] and others [40,45,47], the strength deficits were less substantial. For instance, Ronga et al. [44] reported deficits of 33.7% and 37.3% at angular velocities of 60°/s and 120°/s, respectively, measured with an isokinetic dynamometer approximately three years post-surgery.

Regarding differences between surgical techniques, both reconstruction and repair of the MPFL were investigated in Tompkins et al. [47]. The analysis showed no differences in the two options, with a deficit at 30° in the range of 7.6–7.8%, corresponding to ESs in the range of −0.27 to −0.18. Woods et al. [48] assessed the arthroscopic release of the lateral retinaculum, and data analysis revealed a quadriceps strength deficit of 20.2% with an ES of −0.76.

In the review by Forde et al. [64], lower extremity strength was investigated in patients who had suffered from at least one patellar dislocation and had been treated surgically or non-surgically. This study concluded that strength deficits in the extensor apparatus of the affected knee are frequently observed and may persist in the long term. However, the certainty of this result is limited due to the high variability in the clinical and methodological characteristics and quality of the included studies. The review also examined the strength of the hip muscle complex and knee flexors, which did not appear deficient in these patients, though the same limitations applied. These contradictory results warrant further research, especially in terms of a systematic comparison of the different techniques and approaches to offer guidance in the choice of the more effective treatment.

A comprehensive management framework should be adopted that considers broader aspects of the rehabilitation approach. Further studies are needed to test different approaches for treating this pathology, with larger sample sizes to develop more precise rehabilitation criteria and establish practical guidelines to limit possible deficits. Another important consideration is the measurement of force development, which should be based on standardized outcomes that allow for valid comparisons between different studies and instruments, enabling reliable intra- and inter-study assessments.

Finally, there is a significant gap in the literature regarding the appropriate timing for patients to return to sports. The lack of robust data on surgical and rehabilitation treatments complicates the process of defining readiness for return to sports activities. Current evidence on this topic is sparse and primarily relates to the athletic population [68,69] and healthy adults [70], with the assumption that force differences between limbs in knee extension and flexion should typically be less than 10%. Thus, it is recommended that force symmetry for return to sports be maintained over 85–90% [59]. In the few recent reviews investigating return-to-play criteria for patients who have suffered patellar dislocations, results were heterogeneous, and no clear guidelines have been established. Most studies provided recommendations based on time rather than objective criteria, such as strength measurements or functional tests [50,71]. However, time-based criteria for return to sports may be inappropriate after patellar dislocations. Objective assessments of lower limb strength, particularly quadriceps strength, should be incorporated into rehabilitation protocols to ensure that patients achieve the highest possible limb symmetry index (LSI), improve their quality of life, and make a cautious return to sports [59].

Although no studies have directly investigated the effects of improved lower limb strength in patients who have undergone patellar dislocation, it is reasonable to assume that such improvements could have beneficial effects, as demonstrated in other knee pathologies, such as osteoarthritis, anterior cruciate ligament (ACL) injury, or quadriceps tendon tears [72,73,74]. Further studies and randomized clinical trials are necessary to better understand the most effective approaches, the impact of enhanced lower extremity strength, and the optimal timing for return to sports in patients who have experienced a patellar dislocation.

This systematic review with meta-analysis does have limitations. The clinical and methodological characteristics, strength testing procedures, units of measurement, and instruments used in the included studies were heterogeneous, making it impossible to perform meta-analysis on all the studies and network meta-analysis to rank the various approaches and types of intervention. The different rehabilitation approaches were not always explicitly mentioned, preventing a clear determination of the superiority of one intervention over another. Additionally, in several cases, the number of participants was less than 20. 

However, the strength of this study lies in its meta-analysis of data, which provides a more rigorous evaluation than other reviews on the same topic that did not include statistical analysis. Furthermore, only objective and quantifiable outcomes were evaluated, allowing for comparison with quantitative data from the healthy population or the contralateral uninvolved limb. In the studies included, different modalities and grades were used to test quadriceps muscle strength, and follow-up assessments were conducted to investigate the long-term strength recovery process, with subjects evaluated in the short, medium, and long terms, up to three years.

## 5. Conclusions

The present systematic review and meta-analysis revealed that strength deficits in the quadriceps during knee extension are frequently observed in individuals who have experienced primary or recurrent patellar dislocations, being more pronounced in those treated conservatively. 

Further research is necessary to explore the recovery process after patellar dislocation, taking into account additional factors beyond quadriceps strength and evaluating diverse rehabilitation strategies. Moreover, future studies should emphasize the use of standardized outcome measures, consistent units of measurement, and uniform instrumentation to facilitate data comparison across studies and build robust, consistent evidence.

## Figures and Tables

**Table 1:** Characteristics of the studies included. Abbreviations: mo (months); MPFL (medial patellofemoral ligament), NR (not reported); SD (standard deviation), VM (vastus medialis); and y (years).

Author (Publication Year)	Study Design	Country	Group Description	Type of Intervention	Follow-Up (Mean (SD), Range)	Number of Patients (Male/Female)	Age (Mean (SD), Range)	Number of Patients Who Completed the Follow-Up
Mikashima et al. (2004) [43]	Retrospective study	Japan	Intervention 1 group	Elmslie-Trillat distal realignment procedure	41 (8.7) mo (range 28 to 52 mo)	20 (5/15) with recurrent patellar dislocation and subluxation	26.4 (9.7) y (range 14 to 45 y)	20
			Intervention 2 group	Elmslie-Trillat distal realignment procedure + MPFL reconstruction	31.7 (10.5) mo (range 24 to 44 mo)	20 (6/14) with recurrent patellar dislocation and subluxation	26.0 (10.0) y (range 16 to 55 y)	20
Woods et al. (2006) [48]	Case series (prospective cohort study)	USA	Intervention group (with contralateral limb as control)	Arthroscopic lateral retinacular release, including complete release of the vastus lateralis tendon	27 mo (range 24–43 mo)	24 (7/17) with recurrent patellar dislocation	22.3 y; 26.2 (14.6) y (M); 20.7 (6.6) y (F)	20
Ronga et al. (2009) [44]	Case series (prospective cohort study)	Italy	Intervention group (with contralateral limb as control)	MPFL reconstruction using a hamstring graft passed through two transverse patellar tunnels	37.2 mo (range 30–48 mo) [3.1 y (range, 2.5–4 y)]	28 (21/7) with recurrent patellar dislocation	32.5 (11.4) y; (range 19 to 40 y)	24
Smith et al. (2014) [45]	Observational, non-experimental repeated measures study	UK	Intervention	MPFL reconstruction	Up to 12 mo	30 (16/14) with recurrent patellar dislocation	23.1 (6.4) y	30 at baseline, 27 at 1.5 mo, 21 at 3 mo, 21 at 12 mo
Tompkins et al. (2014) [47]	Retrospective study	USA	Intervention 1 group (with contralateral limb as control)	MPFL reconstruction	29.2 (15.9) mo	11 (NR) with recurrent patellar instability	19.8 y	8 patients (4/4, 1 bilateral), totaling 9 knees
			Intervention 2 (with contralateral limb as control)	MPFL repair	43 (19.9) mo	29 (NR) with recurrent patellar instability	20.1 y	12 patients (3/9, 2 bilateral), totaling 14 knees
Smith et al. (2015) [46]	Pragmatic multi-center RCT	UK	Intervention 1	Strengthening of VM	Up to 12 mo	25 (14/11) with first-time patellar dislocation	23.9 (7.5)	16 at 1.5 mo, 10 at 6 mo, 10 at 12 mo
			Intervention 2 (considered control in the study)	Strengthening of all quadriceps	Up to 12 mo	25 (14/11) with first-time patellar dislocation	23.0 (6.9)	21 at 1.5 mo, 15 at 6 mo, 14 at 12 mo
Asaeda et al. (2016) [40]	Case series with matched controls (case-control study)	Japan	Intervention group	MPFL reconstruction	Up to 12 mo	11 (3/8) with recurrent patellar dislocation	21.2 (7.6) y, range 15 to 35 y	11 pre-operation, 10 at 3 mo, 9 at 6 mo, 11 at 12 mo
			Control group	Uninjured patients	Not applicable	15 (NR)	22.1 (1.8) y	Not applicable
Arrebola et al. (2019) [39]	Cross-sectional, case-control, observational study	Brazil	Intervention group	No intervention	Not applicable	44 (14/30) with at least one episode of atraumatic patellar dislocation	22 (8) y	Not applicable
			Control group	Uninjured patients	Not applicable	44 (11/33)	21 (5) y	Not applicable
Keilani et al. (2019) [41]	Retrospective pilot study	Austria	Intervention 1 group	MPFL reconstruction	47 mo	6 (6/0) with recurrent patellar dislocation	33 y (range 18 to 38 y)	6
			Intervention 2 group	Elmslie-Trillat distal realignment procedure	43 mo	6 (6/0) with recurrent patellar dislocation	26 y (range 19 to 32 y)	6
Lucas et al. (2020) [42]	Cross-sectional study	USA	Intervention group	No intervention	Not applicable	16 (3/13) with recurrent patellar instability	21.1 (4.2) y	Not applicable
			Control group	Uninjured patients	Not applicable	16 (3/13)	21.1 (3.9) y	Not applicable

**Table 2:** Quality Analysis.

Study	Quality Items												
	Q1	Q2	Q3	Q4	Q5	Q6	Q7	Q8	Q9	Q10	Q11	Q12	Q13
Case series studies													
Woods et al. [48]	Yes	Yes	Yes	Yes	Yes	Yes	Yes	Yes	Yes	N/A	-	-	-
Ronga et al. [44]	Yes	Yes	Yes	Yes	Yes	Yes	Yes	Yes	N/A	N/A	-	-	-
Case-control studies													
Mikashima et al. [43]	Yes	N/A	Yes	Yes	Yes	N/A	N/A	Yes	Yes	N/A	-	-	-
Smith TO et al. [45]	N/A	N/A	N/A	Yes	N/A	N/A	N/A	Yes	Yes	Yes	-	-	-
Tompkins et al. [47]	Yes	Yes	Yes	Yes	Yes	N/A	N/A	Yes	Yes	Yes	-	-	-
Asaeda et al. [40]	Yes	Yes	Yes	Yes	Yes	N/A	N/A	Yes	Yes	Yes	-	-	-
Keilani et al. [41]	Yes	Yes	Yes	Yes	Yes	N/A	N/A	Yes	Yes	N/A	-	-	-
RCT													
Smith and Chester et al. [46]	Yes	Yes	Yes	Yes	Yes	Yes	Yes	Yes	Yes	Yes	Yes	Yes	Yes
Cross-sectional studies													
Arrebola et al. [39]	Yes	Yes	Yes	Yes	Yes	N/A	Yes	Yes	-	-	-	-	-
Lucas et al. [42]	Yes	Yes	Yes	Yes	N/A	N/A	Yes	N/A	-	-	-	-	-

**Table 3:** Outcome measurements: strength and torque values are expressed in Newton-meters [N·m], Newton [N], or Newton-meters/kilograms. Abbreviations: IQR (interquartile range); mo (months).

Author	Type of Dynamometer	Outcome Measurements	Residual Deficit/Gain (Absolute and in Percentage)
Mikashima et al. [43]	Mobile	Strength of extension of affected/non-affected knee (% affected/not affected limb)	Intervention 1: −33.2% (quadriceps mean power = 66.8 ± 7.2%) Intervention 2: −24.7% (quadriceps mean power = 75.3 ± 23.3%)
Woods et al. [48]	Isokinetic	Knee extension moment 90°/s [N·m]	Residual deficit of −10.5, −8.5 with respect to the baseline (−20.2%); from 32.3 ± 13.9 pre-operative to 41.5 ± 12.9 at the follow-up in the involved quadriceps; from 51.3 ± 12.8 pre-operative to 52.0 ± 13.5 at the follow-up in the uninvolved quadriceps Gain of 9.2 (28.5%); 32.3 ± 13.9 pre-operation and 41.5 ± 12.9 at the follow-up in the involved quadriceps
Ronga et al. [44]	Isokinetic	Maximum average knee extension torque at 60°/s [N·m]	−60.2 (−33.7%); 118.3 ± 47.8 in the operated limb, 178.5 ± 37.3 in the non-operated limb
		Maximum average knee extension torque at 120°/s [N·m]	−56 (−37.3%); 94 ± 49.7 in the operated limb, 150 ± 31 in the non-operated limb
Smith et al. [45]	Mobile	Isometric knee extension force at 0° [N]	Gain of 25.8 (80.4%); 32.1 ± 14.6 at baseline, 30.1 ± 14.4 at 1.5 mo, 44.2 ± 20.6 at 3 mo, 57.9 ± 24.6 at 12 mo
		Isometric knee extension force at 40° [N]	Gain of 40.7 (91.5%); 44.5 ± 28.6 at baseline, 50.3 ± 28.7 at 1.5 mo, 63.2 ± 41.4 at 3 mo, 85.2 ± 38.8 at 12 mo
		Isometric knee extension force at 80° [N]	Gain of 40.9 (68.1%); 60.1 ± 47.0 at baseline, 69.1 ± 33.3 at 1.5 mo, 88.3 ± 48.7 at 3 mo, 101.0 ± 49.4 at 12 mo
Tompkins et al. [47]	Mobile	Average isometric knee extension torque at 30° [Nm/kg]	Intervention 1: −0.09 (−7.8%); 1.07 [95% CI 0.82–1.32] in the involved side and 1.16 [95% CI 0.92–1.4] in the uninvolved side Intervention 2: −0.09 (−7.6%); 1.09 [95% CI 0.77–1.41] in the involved side and 1.18 [95% CI 0.91–1.45] in the uninvolved side
		Average isometric knee extension torque at 60° [Nm/kg]	Intervention 1: −0.09 (−4.7%); 1.82 [95% CI 1.51–2.13] in the involved side and 1.91 [95% CI 1.43–2.39] in the uninvolved side Intervention 2: −0.36 (−16.6%); 1.81 [95% CI 1.28–2.34] in involved side and 2.17 [95% CI 1.71–2.63] in uninvolved side
Smith et al. [46]	Mobile	Knee extension force at 0° [N]	Intervention 1: gain of 61.9 (172.9%); 35.8 ± 38.9 at baseline, 93.5 ± 47.1 at 1.5 mo, 110.9 (IQR 50.6–159.2) at 6 mo, 91.5 (IQR 75.0–126.5) at 12 mo Intervention 2: gain of 74.1 (221.9%); 33.4 ± 43.8 at baseline, 83.9 ± 38.4 at 1.5 mo, 94.4 (IQR 81.3–143.2) at 6 mo, 102.5 (IQR 83.6–136.5) at 12 mo
		Knee extension force at 30° [N]	Intervention 1: gain of 115.7 (134.7%); 85.9 ± 58.8 at baseline, 167.1 ± 66.5 at 1.5 mo, 177.0 (IQR 124.2–202.4) at 6 mo, 190.4 (IQR 178.2–236.1) at 12 mo Intervention 2: gain of 104.5 (116.2%); 89.9 ± 50.5 at baseline, 164.7 ± 70.2 at 1.5 mo, 170.5 (IQR 136.2–196.4) at 6 mo, 186.6 (IQR 146.1–250.5) at 12 mo
		Knee extension force at 60° [N]	Intervention 1: gain of 120.9 (123.5%); 97.9 ± 48.5 at baseline, 172.0 ± 56.1 at 1.5 mo, 216.9 (IQR 148.9–236.6) at 6 mo, 230.4 (IQR 158.8–267.1) at 12 mo Intervention 2: gain of 105.4 (90.7%); 116.2 ± 65.4 at baseline, 180.3 ± 74.1 at 1.5 mo, 204.5 (IQR 136.3–253.0) at 6 mo, 228.7 (IQR 154.7–281.5) at 12 mo
		Knee extension force at 90° [N]	Intervention 1: gain of 135.0 (146.9%); 100.4 ± 64.9 at baseline, 177.6 ± 63.8) at 1.5 mo, 189.8 (IQR 148.5–237.7) at 6 mo, 247.9 (IQR 178.8–281.1) at 12 mo Intervention 2: 120.8 (102.1%); 118.3 ± 89.2 at baseline, 181.6 ± 75.0 at 1.5 mo, 245.0 (IQR 134.3–267.4) at 6 mo, 258.4 (IQR 172.2–286.6) at 12 mo
Asaeda et al. [40]	Mobile	Strength of extension of affected/non-affected knee (% affected/not affected limb)	−34.3%; 86.4 ± 42.3% pre-operative, 59.8 ± 39.5% at 3 mo, 67.9 ± 23.5% at 6 mo, 52.1 ± 24.3% at 12 mo
Arrebola et al. [39]	Mobile	Quadriceps strength [kgf/kg·100]	−53.44 (−43.7%); 40.44 ± 12.33 in the experimental group and 71.84 ± 14.22 in the control group (Cohen’s d = 2.35) −6.98 (−14.8%); 40.14 ± 12.99 in the affected side and 47.12 ± 12.88 in the non-affected side in a subsample (Cohen’s d = 0.53)
Keilani et al. [41]	Isokinetic	Peak torque at 60°/s knee extension normalized to participant’s body weight [Nm/kg]	Intervention 1: −17% with respect to sex and age-related reference values Intervention 2: −15% with respect to sex and age-related reference values Intervention 1: −16% muscle strength compared to Intervention 2
Lucas et al. [42]	Mobile	Knee extension force [Nm/kg]	−9.3 (−39.1%); 14.5 ± 4.1 in the experimental group and 23.8 ± 7.2 in the control group (Cohen’s d = 1.24)

**Table 4:** Effect sizes (ESs) computed in the meta-analysis.

Study	ES Description	ES	95% CI	*p*-Value
Woods et al. [48]	Residual deficit	−0.76	−1.14 to −0.39	<0.001
	Gain	0.66	0.29 to 1.02	<0.001
Ronga et al. [44]	Residual deficit at 60°/s	−1.31	−1.73 to −0.90	<0.001
	Residual deficit at 120°/s	−1.18	−1.57 to −0.78	<0.001
Smith et al. [45]	Gain at 0°	1.08	0.68 to 1.49	<0.001
	Gain at 40°	1.09	0.68 to 1.50	<0.001
	Gain at 80°	0.81	0.44 to 1.19	<0.001
Tompkins et al. [47]	Residual deficit at 30° in the intervention 1 group	−0.23	−0.71 to 0.26	0.362
	Residual deficit at 30° in the intervention 2 group	−0.16	−0.57 to 0.25	0.456
	Residual deficit at 60° in the intervention 1 group	−0.13	−0.60 to 0.35	0.608
	Residual deficit at 60° in the intervention 2 group	−0.38	−0.80 to 0.05	0.082
Smith et al. [46]	Gain at 0° in the intervention 1 group	1.34	0.71 to 1.98	<0.001
	Gain at 0° in the intervention 2 group	1.60	1.00 to 2.19	<0.001
	Gain at 30° in the intervention 1 group	1.91	1.13 to 2.69	<0.001
	Gain at 30° in the intervention 2 group	1.23	0.71 to 1.74	<0.001
	Gain at 60° in the intervention 1 group	1.25	0.64 to 1.86	<0.001
	Gain at 60° in the intervention 2 group	1.02	0.54 to 1.51	<0.001
	Gain at 90° in the intervention 1 group	1.53	0.85 to 2.20	<0.001
	Gain at 90° in the intervention 2 group	1.24	0.72 to 1.76	<0.001
Asaeda et al. [40]	Residual deficit	−0.80	−1.30 to −0.30	0.002
Arrebola et al. [39]	Residual deficit	−2.34	−2.88 to −1.80	<0.001
Lucas et al. [42]	Residual deficit	−1.55	−2.32 to −0.77	<0.001

## Data Availability

The original contributions presented in the study are included in the article.
